# 1-(2,5-Dimeth­oxy­phen­yl)-3-(2-hy­droxy­eth­yl)urea

**DOI:** 10.1107/S1600536810028436

**Published:** 2010-07-24

**Authors:** Hyeong Choi, Taewoo Lee, Byung Hee Han, Sung Kwon Kang, Chang Keun Sung

**Affiliations:** a Department of Chemistry, Chungnam National University, Daejeon 305-764, Republic of Korea; b Department of Food Science and Technology, Chungnam National University, Daejeon 305-764, Republic of Korea

## Abstract

In the title compound, C_11_H_16_N_2_O_4_, the 2,5-dimeth­oxy­phenyl moiety is almost planar, with an r.m.s. deviation of 0.026 Å. The dihedral angle between the benzene ring and the plane of the urea moiety is 13.86 (5)°. The mol­ecular structure is stabilized by a short intra­molecular N—H⋯O hydrogen bond. In the crystal, inter­molecular N—H⋯O and O—H⋯O hydrogen bonds link the mol­ecules into a three-dimensional network.

## Related literature

For general background, see: Francisco *et al.* (2006 ▶); Jimenez *et al.* (2001 ▶); Korner & Pawelek (1982 ▶); Urabe *et al.* (1998 ▶). For the development of potent inhibitory agents of tyrosinase and melanin formation as whitening agents, see: Battaini *et al.* (2000 ▶); Cabanes *et al.* (1994 ▶); Choi *et al.* (2010 ▶); Germanas *et al.* (2007 ▶); Hong *et al.* (2008 ▶); Kwak *et al.* (2010 ▶); Lemic-Stojcevic *et al.* (1995 ▶); Lee *et al.* (2007 ▶); Liangli (2003 ▶); Thanigaimalai *et al.* (2010 ▶); Yi *et al.* (2009 ▶, 2010 ▶).
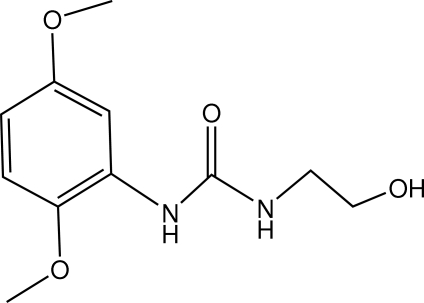


## Experimental

### 

#### Crystal data

C_11_H_16_N_2_O_4_
*M* *_r_* = 240.26
Monoclinic, 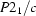

*a* = 10.8571 (9) Å
*b* = 11.5559 (10) Å
*c* = 9.9337 (8) Å
β = 109.514 (4)°
*V* = 1174.73 (17) Å^3^
*Z* = 4
Mo *K*α radiation
μ = 0.10 mm^−1^
*T* = 173 K
0.21 × 0.18 × 0.09 mm

#### Data collection

Bruker SMART CCD area-detector diffractometer
9411 measured reflections
2352 independent reflections
1982 reflections with *I* > 2σ(*I*)
*R*_int_ = 0.062

#### Refinement

*R*[*F* ^2^ > 2σ(*F* ^2^)] = 0.034
*wR*(*F* ^2^) = 0.090
*S* = 1.07
2352 reflections
168 parameters
H atoms treated by a mixture of independent and constrained refinement
Δρ_max_ = 0.19 e Å^−3^
Δρ_min_ = −0.25 e Å^−3^

### 

Data collection: *SMART* (Bruker, 2002 ▶); cell refinement: *SAINT* (Bruker, 2002 ▶); data reduction: *SAINT*; program(s) used to solve structure: *SHELXS97* (Sheldrick, 2008 ▶); program(s) used to refine structure: *SHELXL97* (Sheldrick, 2008 ▶); molecular graphics: *ORTEP-3 for Windows* (Farrugia, 1997 ▶) and *DIAMOND* (Brandenburg, 2010 ▶); software used to prepare material for publication: *WinGX* (Farrugia, 1999 ▶).

## Supplementary Material

Crystal structure: contains datablocks global, I. DOI: 10.1107/S1600536810028436/jh2184sup1.cif

Structure factors: contains datablocks I. DOI: 10.1107/S1600536810028436/jh2184Isup2.hkl

Additional supplementary materials:  crystallographic information; 3D view; checkCIF report

## Figures and Tables

**Table 1:** Hydrogen-bond geometry (Å, °)

*D*—H⋯*A*	*D*—H	H⋯*A*	*D*⋯*A*	*D*—H⋯*A*
N7—H7⋯O14	0.870 (14)	2.153 (13)	2.5995 (12)	111.4 (11)
N7—H7⋯O13^i^	0.870 (14)	2.251 (14)	3.0473 (13)	152.3 (12)
N10—H10⋯O13^i^	0.856 (14)	2.156 (14)	2.9642 (12)	157.1 (12)
O13—H13⋯O9^ii^	0.887 (17)	1.858 (18)	2.7417 (11)	174.0 (15)

Symmetry codes: (i) 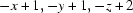; (ii) 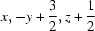.

**Fig. 1.**
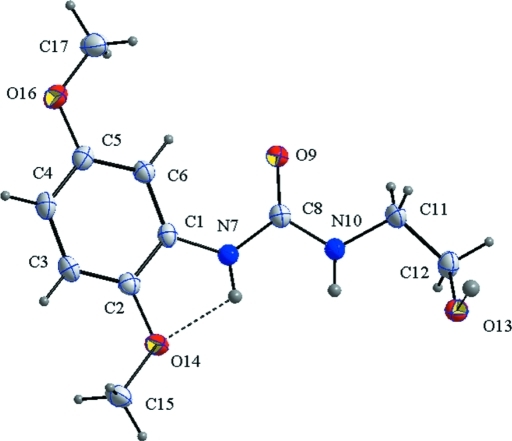
Molecular structure of (l), showing the atom-numbering scheme and 50% probability ellipsoids. Intramolecular N—H···O bond is shown as dashed lines.

**Fig. 2.**
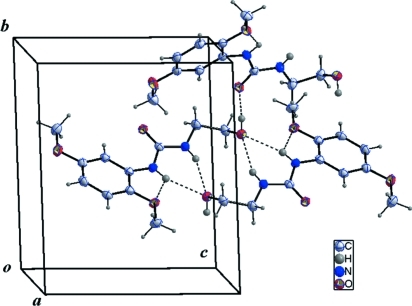
Part of the crystal structure of (I), showing 3-D network of molecules linked by intermolecular N—H···O and O—H···O hydrogen bonds.

## supplementary crystallographic information

### Comment

The melanin production is primarily responsible for the skin color, and melanin plays a vital role in the absorption of free radicals formed in cytoplasm and in protecting human skin from the harmful UV-radiation and from scavenging chemicals (Francisco *et al.*, 2006). Tyrosinase is a multi-functional copper-containing enzyme widely distributed in microorganisms, plants and animals (Jimenez *et al.*, 2001), and it is a key enzyme that catalyzes two distinct reactions of melanin synthesis; the hydroxylation of tyrosine by monophenolase action and the oxidation of *L*-dopa to *o*-dopaquinone by diphenolase action (Korner & Pawelek, 1982). The increased production and accumulation of melanin characterizes a large number of dermatological disorders, which include acquired hyper-pigmentation, causing melasma, freckles, post-inflammatory melanoderma, and solar lentigo (Urabe *et al.*, 1998). Therefore, treatments using potent inhibitory agents on tyrosinase and melanin formation may be cosmetically useful. In recent years, various inhibitors were obtained from natural and synthetic sources with their industrial importance such as azelaic acid (Lemic-Stojcevic *et al.*, 1995), kojic acid (Battaini *et al.*, 2000), albutin (Cabanes *et al.*, 1994), (*R*)-HTCCA (Liangli, 2003) and *N*-phenylthiourea (Thanigaimalai *et al.*, 2010). They contain aromatic, methoxy, hyroxyl (Hong *et al.*, 2008; Lee *et al.*, 2007), aldehyde (Yi *et al.*, 2010), amide (Kwak *et al.*, 2010; Choi *et al.*, 2010), thiosemicarbazone (Yi *et al.*, 2009) and thiazole (Germanas *et al.*, 2007) groups in their structure, and act as a specific functional group to make the skin whiter by inhibiting the production of melanin. However, most of them are not potent enough to put into practical use due to their weak individual activities, poor skin penetration, low stability of formulations, toxicity and/or safety concerns. Consequently, much research is needed to develop novel tyrosinase inhibitors with better activities together with lower side effects. To complement the inadequacy of current whitening agents mentioned above and maximize the inhibition of melanin creation, we have synthesized the title compound, 1-(2,5-dimethoxyphenyl)-3-(2-hydroxyethyl)urea, (I), from the reaction of ethanolamine and 2,5-dimethoxyphenyl isocyanate under ambient condition.

The 2,5-dimethoxyphenyl moiety is almost planar with r.m.s. deviation of 0.026 Å from the corresponding least-squares plane defined by the ten constituent atoms. The dihedral angle between the phenyl ring and the plane of urea moiety is 13.86 (5) °. The molecular structure is stabilized by a short intramolecular N7—H7···O14 hydrogen bond (Fig. 1). In the crystal, intermolecular N—H···O and O—H···O hydrogen bonds link the molecules into a three-dimensional network (Fig. 2).

### Experimental

The ethanolamine and 2,5-dimethoxyphenyl isocyanate were purchased from Sigma Chemical Co. Solvents used for organic synthesis were redistilled before use. All other chemicals and solvents were of analytical grade and were used without further purification. The title compound (I) was prepared from the reaction of ethanolamine (0.1 ml, 2 mmol) with 2,5-dimethoxyphenyl isocyanate (0.5 g, 3 mmol) in acetonitrile (6 ml). The reaction was completed within 10 min at room temperature. The reaction mixture was filtered rapidly with ether. Removal of the solvent gave a white solid (90% m.p. 419 K). Single crystals were obtained by slow evaporation of the ethanol at room temperature.

### Refinement

The H atoms of the NH and OH groups were located in a difference Fourier map and refined freely. The remaining H atoms were positioned geometrically and refined using a riding model with C—H = 0.93–0.97 Å, and with *U*_iso_(H) = 1.2*U*_eq_ (C) for aromatic and metylene, and 1.5*U*_eq_(C) for methyl H atoms.

### Crystal data

**Table:**

C_11_H_16_N_2_O_4_	*F*(000) = 512
*M**_r_* = 240.26	*D*_x_ = 1.358 Mg m^−^^3^
Monoclinic, *P*2_1_/*c*	Mo *K*α radiation, λ = 0.71073 Å
Hall symbol: -P 2ybc	Cell parameters from 4246 reflections
*a* = 10.8571 (9) Å	θ = 2.8–28.2°
*b* = 11.5559 (10) Å	µ = 0.10 mm^−^^1^
*c* = 9.9337 (8) Å	*T* = 173 K
β = 109.514 (4)°	Block, colourless
*V* = 1174.73 (17) Å^3^	0.21 × 0.18 × 0.09 mm
*Z* = 4	

### Data collection

**Table:**

Bruker SMART CCD area-detector diffractometer	*R*_int_ = 0.062
φ and ω scans	θ_max_ = 26.5°, θ_min_ = 2.7°
9411 measured reflections	*h* = −6→13
2352 independent reflections	*k* = −14→9
1982 reflections with *I* > 2σ(*I*)	*l* = −12→7

### Refinement

**Table:**

Refinement on *F*^2^	0 restraints
Least-squares matrix: full	H atoms treated by a mixture of independent and constrained refinement
*R*[*F*^2^ > 2σ(*F*^2^)] = 0.034	*w* = 1/[σ^2^(*F*_o_^2^) + (0.0526*P*)^2^] where *P* = (*F*_o_^2^ + 2*F*_c_^2^)/3
*wR*(*F*^2^) = 0.090	(Δ/σ)_max_ < 0.001
*S* = 1.07	Δρ_max_ = 0.19 e Å^−^^3^
2352 reflections	Δρ_min_ = −0.25 e Å^−^^3^
168 parameters	

### Special details

**Table:**

Geometry. All e.s.d.'s (except the e.s.d. in the dihedral angle between two l.s. planes) are estimated using the full covariance matrix. The cell e.s.d.'s are taken into account individually in the estimation of e.s.d.'s in distances, angles and torsion angles; correlations between e.s.d.'s in cell parameters are only used when they are defined by crystal symmetry. An approximate (isotropic) treatment of cell e.s.d.'s is used for estimating e.s.d.'s involving l.s. planes.

### Fractional atomic coordinates and isotropic or equivalent isotropic displacement parameters (Å^2^)

**Table:**

	*x*	*y*	*z*	*U*_iso_*/*U*_eq_	
C1	0.26322 (10)	0.50237 (9)	0.48181 (12)	0.0208 (2)	
C2	0.15431 (11)	0.43224 (9)	0.47055 (12)	0.0220 (3)	
C3	0.05312 (11)	0.42372 (9)	0.34222 (12)	0.0252 (3)	
H3	−0.019	0.3778	0.3357	0.03*	
C4	0.05831 (11)	0.48333 (9)	0.22288 (13)	0.0264 (3)	
H4	−0.0101	0.4778	0.1366	0.032*	
C5	0.16604 (11)	0.55102 (9)	0.23333 (12)	0.0242 (3)	
C6	0.26888 (11)	0.56150 (9)	0.36195 (12)	0.0228 (3)	
H6	0.3407	0.6076	0.3677	0.027*	
N7	0.36023 (9)	0.50709 (8)	0.61700 (10)	0.0234 (2)	
H7	0.3458 (12)	0.4616 (12)	0.6799 (15)	0.035 (4)*	
C8	0.45894 (10)	0.58708 (9)	0.66522 (12)	0.0205 (2)	
O9	0.48440 (8)	0.65994 (6)	0.58761 (8)	0.0260 (2)	
N10	0.52601 (9)	0.57919 (8)	0.80586 (11)	0.0250 (2)	
H10	0.5012 (13)	0.5293 (11)	0.8553 (15)	0.033 (3)*	
C11	0.62695 (11)	0.66229 (10)	0.87792 (12)	0.0268 (3)	
H11A	0.5892	0.7391	0.8696	0.032*	
H11B	0.6936	0.6631	0.8329	0.032*	
C12	0.68773 (11)	0.63093 (10)	1.03317 (13)	0.0271 (3)	
H12A	0.7315	0.5568	1.0407	0.032*	
H12B	0.7531	0.6884	1.0801	0.032*	
O13	0.59367 (8)	0.62435 (7)	1.10450 (9)	0.0260 (2)	
H13	0.5628 (15)	0.6954 (15)	1.1048 (16)	0.054 (5)*	
O14	0.15906 (8)	0.37701 (6)	0.59484 (9)	0.0274 (2)	
C15	0.05328 (12)	0.30175 (10)	0.58780 (14)	0.0312 (3)	
H15A	−0.0265	0.3454	0.5619	0.047*	
H15B	0.0686	0.2662	0.6793	0.047*	
H15C	0.0465	0.2429	0.5175	0.047*	
O16	0.16393 (8)	0.60560 (7)	0.10891 (9)	0.0329 (2)	
C17	0.26678 (12)	0.68455 (11)	0.11745 (14)	0.0343 (3)	
H17A	0.267	0.7454	0.1833	0.051*	
H17B	0.2539	0.717	0.0248	0.051*	
H17C	0.3489	0.6445	0.15	0.051*	

### Atomic displacement parameters (Å^2^)

**Table:**

	*U*^11^	*U*^22^	*U*^33^	*U*^12^	*U*^13^	*U*^23^
C1	0.0197 (5)	0.0189 (5)	0.0216 (6)	0.0014 (4)	0.0039 (5)	−0.0032 (4)
C2	0.0235 (6)	0.0185 (5)	0.0240 (6)	0.0011 (4)	0.0078 (5)	−0.0006 (4)
C3	0.0214 (6)	0.0238 (6)	0.0283 (7)	−0.0040 (5)	0.0057 (5)	−0.0044 (5)
C4	0.0232 (6)	0.0288 (6)	0.0226 (7)	−0.0007 (5)	0.0013 (5)	−0.0040 (5)
C5	0.0259 (6)	0.0244 (6)	0.0207 (6)	0.0029 (5)	0.0058 (5)	−0.0004 (4)
C6	0.0213 (6)	0.0227 (5)	0.0234 (6)	−0.0017 (4)	0.0063 (5)	−0.0010 (5)
N7	0.0239 (5)	0.0238 (5)	0.0197 (5)	−0.0049 (4)	0.0034 (5)	0.0027 (4)
C8	0.0192 (6)	0.0200 (5)	0.0215 (6)	0.0012 (4)	0.0058 (5)	−0.0016 (4)
O9	0.0280 (5)	0.0256 (4)	0.0225 (5)	−0.0062 (3)	0.0061 (4)	0.0023 (3)
N10	0.0258 (5)	0.0261 (5)	0.0200 (6)	−0.0075 (4)	0.0034 (5)	0.0014 (4)
C11	0.0240 (6)	0.0309 (6)	0.0237 (7)	−0.0082 (5)	0.0054 (5)	−0.0030 (5)
C12	0.0223 (6)	0.0321 (6)	0.0241 (7)	−0.0023 (5)	0.0042 (5)	−0.0035 (5)
O13	0.0307 (5)	0.0228 (4)	0.0247 (5)	−0.0005 (3)	0.0095 (4)	0.0010 (3)
O14	0.0259 (4)	0.0278 (4)	0.0264 (5)	−0.0069 (3)	0.0060 (4)	0.0041 (3)
C15	0.0289 (6)	0.0268 (6)	0.0378 (7)	−0.0076 (5)	0.0111 (6)	0.0028 (5)
O16	0.0316 (5)	0.0406 (5)	0.0220 (5)	−0.0059 (4)	0.0031 (4)	0.0058 (4)
C17	0.0330 (7)	0.0360 (7)	0.0326 (7)	−0.0024 (5)	0.0092 (6)	0.0094 (6)

### Geometric parameters (Å, °)

**Table:**

C1—C6	1.3918 (15)	N10—H10	0.856 (14)
C1—N7	1.4039 (15)	C11—C12	1.5051 (16)
C1—C2	1.4068 (15)	C11—H11A	0.97
C2—O14	1.3754 (13)	C11—H11B	0.97
C2—C3	1.3806 (17)	C12—O13	1.4262 (13)
C3—C4	1.3883 (16)	C12—H12A	0.97
C3—H3	0.93	C12—H12B	0.97
C4—C5	1.3819 (16)	O13—H13	0.887 (17)
C4—H4	0.93	O14—C15	1.4236 (13)
C5—O16	1.3809 (13)	C15—H15A	0.96
C5—C6	1.3931 (17)	C15—H15B	0.96
C6—H6	0.93	C15—H15C	0.96
N7—C8	1.3746 (14)	O16—C17	1.4224 (14)
N7—H7	0.870 (14)	C17—H17A	0.96
C8—O9	1.2337 (12)	C17—H17B	0.96
C8—N10	1.3457 (15)	C17—H17C	0.96
N10—C11	1.4531 (14)		
			
C6—C1—N7	124.46 (10)	N10—C11—C12	110.24 (9)
C6—C1—C2	119.40 (10)	N10—C11—H11A	109.6
N7—C1—C2	116.14 (10)	C12—C11—H11A	109.6
O14—C2—C3	125.13 (9)	N10—C11—H11B	109.6
O14—C2—C1	114.68 (10)	C12—C11—H11B	109.6
C3—C2—C1	120.19 (10)	H11A—C11—H11B	108.1
C2—C3—C4	120.42 (10)	O13—C12—C11	112.35 (10)
C2—C3—H3	119.8	O13—C12—H12A	109.1
C4—C3—H3	119.8	C11—C12—H12A	109.1
C5—C4—C3	119.44 (11)	O13—C12—H12B	109.1
C5—C4—H4	120.3	C11—C12—H12B	109.1
C3—C4—H4	120.3	H12A—C12—H12B	107.9
O16—C5—C4	115.51 (10)	C12—O13—H13	106.6 (10)
O16—C5—C6	123.35 (10)	C2—O14—C15	116.85 (9)
C4—C5—C6	121.13 (10)	O14—C15—H15A	109.5
C1—C6—C5	119.41 (10)	O14—C15—H15B	109.5
C1—C6—H6	120.3	H15A—C15—H15B	109.5
C5—C6—H6	120.3	O14—C15—H15C	109.5
C8—N7—C1	127.44 (9)	H15A—C15—H15C	109.5
C8—N7—H7	117.6 (9)	H15B—C15—H15C	109.5
C1—N7—H7	114.0 (9)	C5—O16—C17	117.49 (9)
O9—C8—N10	122.59 (10)	O16—C17—H17A	109.5
O9—C8—N7	123.54 (10)	O16—C17—H17B	109.5
N10—C8—N7	113.87 (9)	H17A—C17—H17B	109.5
C8—N10—C11	121.61 (9)	O16—C17—H17C	109.5
C8—N10—H10	118.4 (9)	H17A—C17—H17C	109.5
C11—N10—H10	119.5 (9)	H17B—C17—H17C	109.5

### Hydrogen-bond geometry (Å, °)

**Table:**

*D*—H···*A*	*D*—H	H···*A*	*D*···*A*	*D*—H···*A*
N7—H7···O14	0.870 (14)	2.153 (13)	2.5995 (12)	111.4 (11)
N7—H7···O13^i^	0.870 (14)	2.251 (14)	3.0473 (13)	152.3 (12)
N10—H10···O13^i^	0.856 (14)	2.156 (14)	2.9642 (12)	157.1 (12)
O13—H13···O9^ii^	0.887 (17)	1.858 (18)	2.7417 (11)	174.0 (15)

Symmetry codes: (i) −*x*+1, −*y*+1, −*z*+2; (ii) *x*, −*y*+3/2, *z*+1/2.
